# Comparison of clinicopathological characteristics and survival outcomes between solitary and multiple rectal neuroendocrine tumors: a propensity score-matched study

**DOI:** 10.3389/fonc.2025.1599022

**Published:** 2025-06-05

**Authors:** Ye Zheng, Limei Wang, Jing Guo, Peng Wang, Rui Ji, Jun Liu

**Affiliations:** ^1^ Department of Gastroenterology, Shandong University Qilu Hospital, Jinan, Shandong, China; ^2^ Shandong Provincial Clinical Research Center for Digestive Disease, Shandong University Qilu Hospital, Jinan, Shandong, China

**Keywords:** multiple, rectal neuroendocrine tumor, endoscopic submucosal dissection, clinicopathological characteristics, survival

## Abstract

**Introduction:**

Multiple rectal neuroendocrine tumors (RNETs) are rare rectal malignancies, and there is no consensus on their characteristics and treatments. This study aimed to explore the heterogeneity of key morphological parameters in multiple RNETs and to compare the clinicopathological characteristics between multiple and solitary RNETs.

**Methods:**

A total of 15 patients with multiple RNETs and 89 patients with solitary RNETs treated between 2013 and 2024 were retrospectively analyzed using propensity match analysis to determine their clinicopathological characteristics. WHO grade, the expression of basal diagnostic markers (synaptophysin/chromogranin A/CD56), and somatostatin receptor 2 (SSTR2) were analyzed. Disease-free survival rates were calculated using the Kaplan–Meier method.

**Results:**

Multifocal RNETs were characterized by homogeneous WHO grading (93.3%) and concordant SSTR2 expression. The solitary RNETs group had a significantly higher SSTR2 positivity rate (*p* < 0.05) but significantly lower chromogranin A positivity rate than the multiple RNETs group (*p* < 0.05).

**Conclusion:**

Multiple RNETs demonstrate remarkable homogeneity in core diagnostic parameters. However, compared to solitary RNETs, multifocal presentations exhibit a significantly higher propensity for metastasis/recurrence, warranting intensified therapeutic protocols and enhanced clinicopathological surveillance paradigms.

## 1 Introduction

Rectal neuroendocrine tumors (RNETs) are malignant neoplasms arising from enterochromaffin cells of the distal gastrointestinal tract. RNETs have demonstrated a marked epidemiological surge in recent decades, potentially due to the increased use of colonoscopy (1). RNETs account for 12%–27% of gastrointestinal NETs, and the rectum represents the second most frequent anatomical site of origin (2). While the majority present as solitary lesions, multifocal RNETs constitute a rare clinical entity with reported incidence rates of 2%–5.7% (3). Although multiple RNETs are rare, they are associated with poorer outcomes, including increased lymph node involvement and decreased overall survival, compared to solitary NETs (3–5).

Despite these clinical implications, standardized management protocols for multifocal RNETs remain undefined, with current guidelines extrapolated from solitary tumor data. Given the inherent heterogeneity of neuroendocrine tumors, it is unclear whether this variability is also reflected in multiple primaries, particularly in key diagnostic parameters, such as tumor grade and neuroendocrine markers, and therapeutic targets like somatostatin receptor 2 (SSTR2) expression, and how these findings influence current diagnostic approaches for multiple RNETs. This uncertainty complicates clinical decision-making, as comprehensive immunohistochemical profiling of all lesions is resource-intensive and lacks well-established clinical rationale.

The aim of this study was to investigate the variability of crucial morphological and diagnostic indicator features within multiple RNETs and to compare the clinicopathological characteristics between multiple and solitary RNETs.

## 2 Methodology

### 2.1 Patients and lesions

This retrospective cohort study enrolled patients with pathologically confirmed RNETs undergoing endoscopic resection between 2013 and 2024 at the Department of Gastroenterology at Qilu Hospital of Shandong University. Patients were excluded if they had neuroendocrine carcinomas or incomplete clinicopathological documentation. Institutional Review Board approval for this retrospective report was obtained from the ethics committees of Qilu Hospital, Shandong University (No. KYLL-202306-009). Informed consent was obtained from all participants. In our department, all RNETs scheduled for endoscopic submucosal dissection (ESD) treatment were first evaluated for size via endoscopy and for depth and staging via endoscopic ultrasound (EUS). Before ESD, patients routinely undergo radiological imaging examinations such as CT or MRI to rule out lymph node or distant metastasis. Patients who met the criteria for endoscopic resection (size smaller than 2 cm, EUS stage limited to the T1 stage, and without radiologically detected lymph node or distant metastasis) underwent ESD treatment (6). The lesions were completely removed and sent for pathological examination.

### 2.2 Clinicopathological data collection

Clinical, pathological, and endoscopic data were retrospectively collected from the inpatient medical record system of the Department of Gastroenterology at Qilu Hospital of Shandong University and the Medikang Digital Gastrointestinal Endoscopy Workstation. Demographic parameters, including gender, age, body mass index (BMI), smoking, and alcohol consumption, were systematically documented. Clinicopathological characteristics such as tumor size, number of lesions, anatomical localization, endoscopic resection method, and histopathological characteristics, as well as treatments, were also recorded. Tumor size was determined through histopathological measurement.

Endoscopic specimens were evaluated by pathologists, and immunohistochemical staining and special staining were performed to confirm the tumor size, the involvement of the excised margins, depth of invasion, and the presence of lymphovascular invasion. Each tumor was graded based on its Ki-67 index according to the WHO criteria (G1, <3%; G2, 3%–20%; and G3, >20%). Histologically complete (R0) resection was defined as the microscopic absence of tumor cells at the resection margin. Incomplete resection (R1) was defined as the presence of tumor tissue in the resection margin (vertical or lateral). Indeterminate resection (Rx) was defined as a margin status that could not be assessed because of electrocautery artifacts and inappropriate orientation or fragmentation. For standard diagnostic markers, NETs were classified as positive if they were detected in clumps in the majority of cells, while NETs expressed in scattered single cells (<5%) were classified as focally positive/negative (further referred to as negative).

SSTR2 expression was initially classified into four categories according to the HER2 scoring scheme as proposed by Kasajima et al. (7). For further analyses, all tumors with an SSTR2 2+/3+ score were assigned to the SSTR2-positive category, while SSTR2 1+ and completely negative NETs were assigned to the SSTR2-negative category. During routine clinicopathological examination post-tumor resection, the most locally advanced tumor was identified as the “leading primary NET” and was evaluated regarding its Ki-67 index in order to determine the tumor grade according to WHO criteria and the size of lesions, referring to the definitions in previous literature (8). The leading primary NET served as the reference for our comparative analyses of all multiple tumors.

### 2.3 Follow-up

The follow-up strategy was determined based on the pathological grading and the status of the resection margins. Endoscopy and radiological follow-ups were recommended based on the stages and grades of the lesions. In our center, for RNETs smaller than 1 cm, no follow-up was recommended; for those that are 1 to <2 cm, G2 tumor, or Rx resection, endoscopy and radiological follow-ups at 6 and 12 months were recommended, then as clinically indicated. Among the cases pathologically evaluated as R1 resection or lymphovascular invasion, additional surgery, including lymph node dissection or other salvage treatments, was recommended. The follow-up time was from endoscopic treatment to the latest outpatient visit. The follow-up results were based on the latest outpatient medical record or electronic examination report. Local recurrence was defined as NETs diagnosed at the same site 6 months after the initial resection, whereas metachronous lesions were defined as NETs detected at a site different from the initial rectal tumor 6 months after the initial resection. Disease-free survival (DFS) was defined as the duration from tumor resection to locoregional recurrence, developing metachronous lesions, or distant metastasis.

### 2.4 Statistical analysis

Normally distributed continuous variables are presented as the mean with standard deviation (SD) and compared using Student’s t-test. Non-normally distributed continuous variables were reported as median with an interquartile range (IQR) and compared using the Mann–Whitney U test. Categorical variables were compared using the χ^2^ test or Fisher’s exact test.

Propensity score matching (PSM) was adopted for comparing the disease-free survival analysis between the solitary and multiple rectal neuroendocrine groups and to minimize selection bias. The matching ratio was 4:1, and the caliper value was set to 0.05. The nearest-neighbor method was used for PSM analysis. Survival curves were generated using the Kaplan–Meier method and were compared using the log-rank test. A two-tailed *p*-value <0.05 was considered statistically significant. All statistical analyses were conducted using SPSS 25.0 (SPSS Inc., Chicago, IL, USA) and R version 4.4.0 (R Foundation for Statistical Computing, Vienna, Austria).

## 3 Results

### 3.1 Patients and lesions

A total of 205 patients were considered for inclusion in this study, including 189 patients with solitary RNETs (group A) and 16 patients with multiple RNETs (group B). However, 100 patients in group A and one patient in group B were excluded because of incomplete clinicopathological data. Finally, 15 patients with multiple RNETs and 89 patients with single RNETs were included in the study. The multifocal group contained 35 lesions, including 12 patients with two lesions, two with three lesions, and one with five lesions. After preoperative endoscopic evaluation, all lesions were found to be smaller than 2 cm. Ultrasonography showed that all lesions were confined to the T1 stage, which met the indications for endoscopic treatment. All patients underwent ESD as the primary treatment (**Table 1**).

**Table 1:** Incidence of multiple lesions in rectal neuroendocrine tumor patients.

No. of tumors	No. of patients	No. of lesions
2	12	24
3	2	6
5	1	5
Total	15	35

### 3.2 Clinicopathological characteristics of multiple RNETs

Endoscopic evaluation revealed predominantly flat-type submucosal yellowish-white protrusions (80%) without ulceration. Red vascular patterns were observed in 6.7% (1/15) of cases.

**Figure 1:**
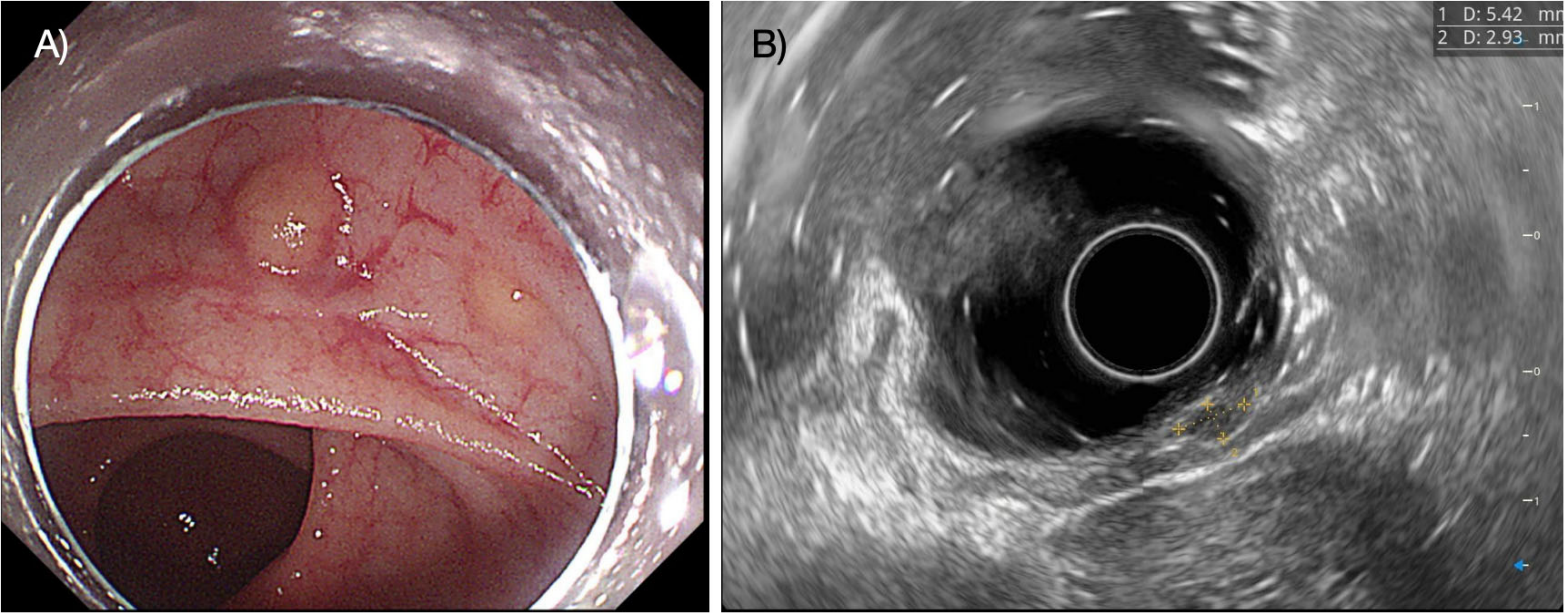
The figure shows two flat, yellowish lesions in the rectum **(A)**, and endoscopic ultrasound reveals that the lesions are located in the submucosal layer **(B)**.

The median age of the patients was 50 years (IQR, 44.0–53.5). Ten patients were male (66.7%), and five patients were female. The median tumor size was 0.6 cm (IQR, 0.5–0.7 cm). Macroscopically, 80% of the tumors were of the flat type. Most tumor invasion was confined to the submucosal layer in all patients, and no lymphovascular invasion was identified (**Figure 1**). More detailed clinicopathological characteristics of the multiple rectal NETs are presented in **Table 2**.

**Table 2:** Clinicopathological characteristics of solitary rectal neuroendocrine tumors (group A) and multiple rectal neuroendocrine tumors (group B).

Variable	Before PSM			After PSM		
	A (n = 89)	B (n = 15)	*p*	A (n = 32)	B (n = 14)	*p*
Age, years, M (IQR)	51.0 (45.0–59.0)	50.0 (44.0–53.5)	0.338	53.5 (43.0–62.0)	51.0 (45.0–55.0)	0.610
Gender, n (%)			0.885			0.624
Female	28 (31.5)	5 (33.3)		7 (21.9)	4 (28.6)	
Male	61 (68.5)	10 (66.7)		25 (78.1)	10 (71.4)	
BMI, kg/m^2^, mean ± SD	25.2 ± 3.6	25.1 ± 2.9	0.962	24.7 ± 3.4	24.6 ± 3.3	0.986
Smoker, n (%)			0.774			0.748
Never	61 (68.5)	11 (73.3)		21 (65.6)	10 (71.4)	
Current and past	28 (31.5)	4 (26.7)		11 (34.4)	4 (28.6)	
Drinker, n (%)			0.514			0.093
Never	61 (68.5)	9 (60.0)		20 (62.5)	5 (35.7)	
Current and past	28 (31.5)	6 (40.0)		12 (37.5)	9 (64.3)	
Size, cm, M (IQR)	0.7 (0.5–0.8)	0.6 (0.5–0.7)	0.262	0.7 (0.5–0.8)	0.6 (0.5–0.7)	0.359
Location, cm, M (IQR) (distance from AV)	6.0 (5.0–8.0)	7.0 (5.0–9.0)	0.247	6.0 (5.0–8.0)	7.0 (5.0–10.0)	0.156
Morphology, n (%)			0.005			0.556
Protruded	42 (47.2)	7 (20.0)		10 (31.3)	5 (23.8)	
Flat	47 (52.8)	28 (80.0)		22 (68.7)	16 (76.2)	
Tumor grade, n (%)			1.000			0.249
G1	82 (92.1)	32 (91.4)		28 (87.5)	21 (100.0)	
G2	7 (7.9)	3 (8.6)		4 (12.5)	0 (0.0)	

AV, anal verge; BMI, body mass index; IQR, interquartile range; M, median; PSM, propensity score matching.

### 3.3 Pathological, immunohistochemical, and special staining findings

#### 3.3.1 Comparative analysis of WHO grade in multiple RNETs

**Figure 2:**
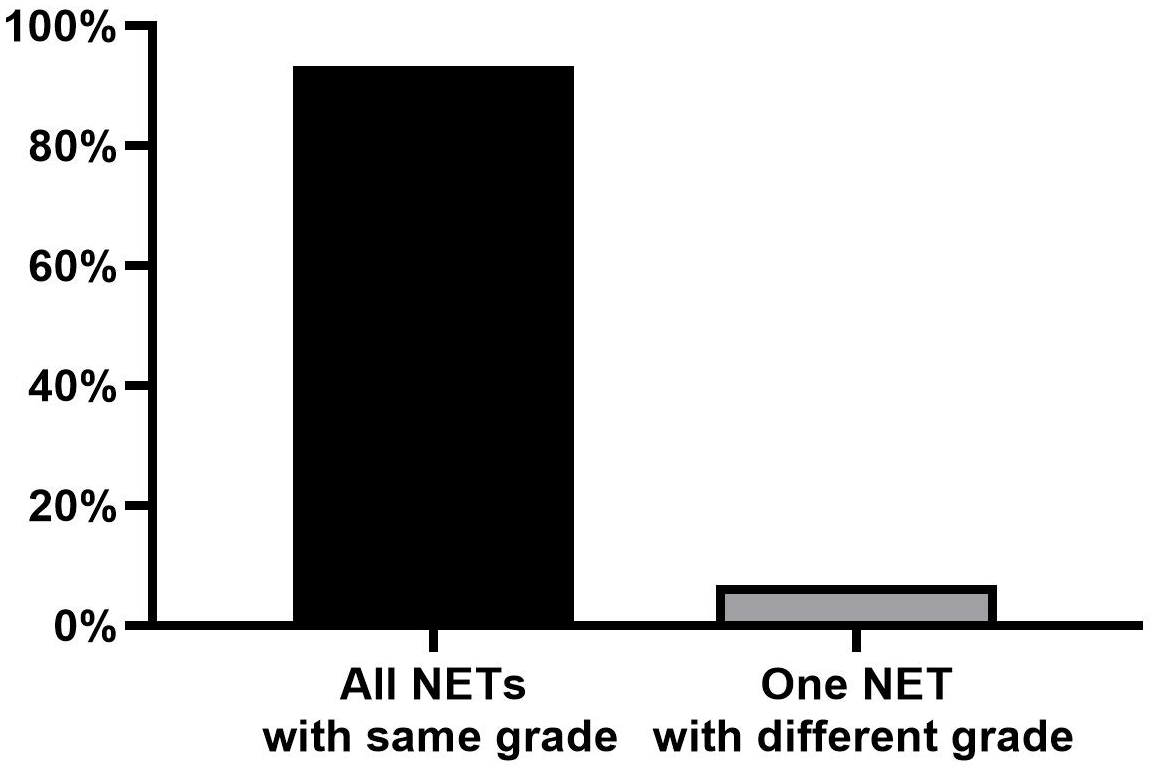
Number of divergent NETs compared to the leading primary NETs for tumor grade. NETs, neuroendocrine tumors.

The majority of tumors (32/35; 91.4%) were classified as G1 based on the Ki-67 index, while three tumors (3/35, 8.6%) were classified as G2. Among the leading primary NETs, 13 of 15 (86.7%) were G1, while two (13.3%) were G2. All synchronous NETs exhibited the same grade (G1, n = 13; G2, n = 1) in 93.3% (14/15) of all patients (**Figure 2**). Only one patient demonstrated a discrepancy, where a G2 leading primary NET was accompanied by smaller synchronous G1 NETs.

#### 3.3.2 Expression of standard diagnostic markers in multiple RNETs

Syn and CD56 were diffusely expressed in all tumors. Among the 15 leading primary NETs, nine (60.0%) were positive for chromogranin A (CgA), while six (40.0%) were negative. The overall CgA positivity rate was 51.4% in all lesions. The expression of Syn, CgA, and CD56 did not vary between the leading primary NETs and the synchronous NETs.

#### 3.3.3 SSTR2 expression in multiple RNETs

Ten of 15 (66.7%) of the leading primary NETs were categorized as SSTR2-positive, and five (33.3%) were SSTR2-negative. The overall SSTR2 positivity rate was 65.7% in all lesions. No difference in SSTR2 expression was noted between the leading primary NET and the smaller synchronous NETs.

### 3.4 Characteristics of solitary and multiple RNETs

The clinical differences between the groups are summarized in **Table 2**. Macroscopically, group B was more likely to contain flat-type tumors compared to group A (80.0% vs. 52.8%, *p* < 0.05). However, there were no significant differences with respect to the presence of solitary and multiple tumors in other features. In the matched cohort (Group A, n = 32; Group B, n = 14), the selected variables were balanced across the two groups (**Table 2**). In group B, 21 lesions were identified among 14 patients.

### 3.5 Comparison of prognostic factors associated with solitary and multiple rectal tumors

**Table 3** summarizes the prognostic factors in the matched solitary and multiple NET groups. The SSTR2 positivity rate was significantly higher in group A compared to group B (84.4% vs. 57.1%, *p* < 0.05). Conversely, the positivity rate of CgA was significantly higher in group B than in group A (57.1% vs. 21.9%, *p* < 0.05). However, there were no significant differences in platelet-to-lymphocyte ratio (PLR) and neutrophil-to-lymphocyte ratio (NLR) between the two groups (*p* > 0.05).

**Table 3:** Comparison of prognostic factors associated with solitary (group A) and multiple (group B) rectal neuroendocrine tumors.

Prognostic Factors	Before PSM		*p*	After PSM		*p*
	A (n = 89)	B (n = 15)		A (n = 32)	B (n = 14)	
NLR, M (IQR)	2.0 (1.4–2.5)	2.2 (1.4–2.9)	0.239	2.0 (1.5–2.6)	2.4 (1.6–3.0)	0.076
PLR, M (IQR)	134.1 (110.5–162.7)	128.87 (92.0–186.8)	0.735	131.5 (118.2–146.7)	145.2 (97.6–189.8)	0.484
CgA, n (%)			<0.001			0.009
Negative	76 (85.4)	17 (48.6)		25 (78.1)	9 (42.9)	
Positive	13 (14.6)	18 (51.4)		7 (21.9)	12 (57.1)	
SSTR2, n (%)			0.100			0.028
Negative	18 (20.2)	12 (34.3)		5 (15.6)	9 (42.9)	
Positive	71 (79.8)	23 (65.7)		27 (84.4)	12 (57.1)	

NLR, neutrophil-to-lymphocyte ratio; PLR, platelet-to-lymphocyte ratio.

### 3.6 ESD resection outcomes of multiple RNETs

The histological complete resection rate (R0) of multiple RNETs was 68.6% (24/35), 11.4% (4/35) R1 resection (two lesions with positive horizontal margins and two with positive vertical margins), and 20% (7/35) Rx resection. As for the safety of ESD procedures, none of the 15 patients in group B had intraoperative complications, including bleeding or perforation.

### 3.7 Clinical outcomes during follow-up

The overall median follow-up period was 20 months (range, 3–60). None of the patients in group A developed recurrence or metastasis. One of the 15 patients in group B developed local recurrence with local lymph node metastasis (LNM). This patient had two tumors (G1 and G2), each measuring less than 10 mm in diameter, with Rx margins and no muscular invasion. After endoscopic resection, the patient was treated with growth inhibitor analogs.

**Figure 3:**
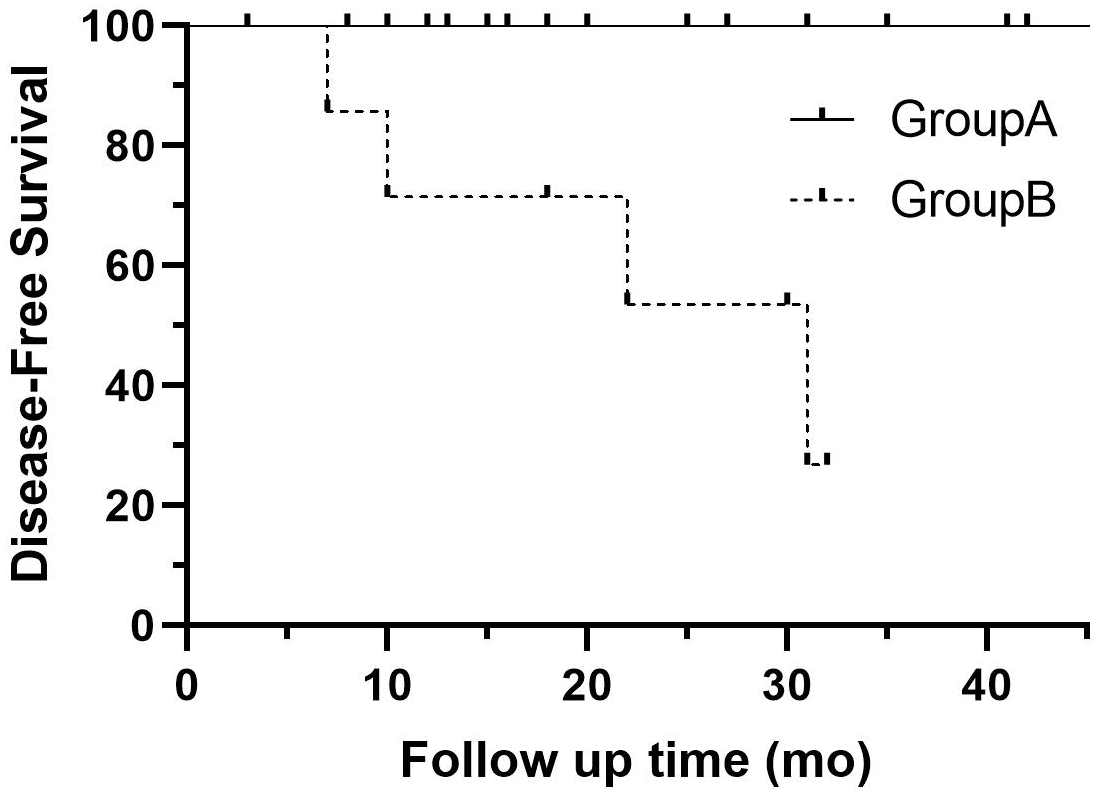
Disease-free survival curve for patients with single RNETs (group A: solid line) and those with multiple RNETs (group B: dotted line). RNETs, rectal neuroendocrine tumors.

Additionally, metachronous rectal NETs were diagnosed in two patients with both G1 lesions, one of whom also had local LNM. The patient with LNM did not receive further treatment or evaluation. One patient with postoperative pathology showing G2 in all lesions and R0 margins has undergone three follow-up colonoscopies to date, none of which indicated recurrence or metachronous lesions. However, somatostatin receptor scintigraphy revealed multiple bone concentrations suggestive of bone metastasis, and the patient was subsequently treated with somatostatin analogs. The clinicopathological characteristics of these patients are detailed in **Table 4**. There was a significant difference in DFS between the two groups (*p* = 0.0003) (**Figure 3**).

**Table 4:** Clinicopathological characteristics of patients who developed recurrence, metastasis, or metachronous rectal neuroendocrine tumors.

No.	Depth of invasion	Resection margin	Grade	Ki-67	Lympho-vascular invasion	Types of recurrence or metastasis	Mode of initial treatment	Salvage treatment	Time interval (months)
1	Submucosal	R0, R0	2, 2	4%, 3%	No	Metastasis	ESD	No	31
2	Submucosal	R0, R0	1, 1	1%, 1%	No	Metachronous	ESD	No	22
3	Submucosal	R0, Rx	1, 1	1%, 1%	No	Metachronous	ESD	No	6
4	Submucosal	Rx, Rx	1, 2	1%, 5%	No	Recurrence	ESD	No	16

R0, complete resection; R1, incomplete resection; Rx, indeterminate resection.

## 4 Discussion

Previous research has indicated that immunohistochemistry is not required for all multifocal jejunoileal neuroendocrine tumors (8, 9). The primary aim of this study was to assess whether immunohistochemistry should be systematically applied to all multiple RNETs, considering the balance between efficiency, cost-effectiveness, accuracy, and optimal patient care. Our findings revealed that tumor grade is consistent in approximately 93.3% of multiple RNETs. Only one case exhibited inconsistency in synchronous primaries, where the routine pathology of the “leading primary NET” was G2 with a smaller G1 lesion. This observation is consistent with previous studies indicating a correlation between tumor size and grade (10). Despite minor discrepancies in synchronous primaries, assessing the grade of the largest RNET was sufficient to accurately represent the overall proliferative activity in multiple RNETs.

Somatostatin exerts inhibitory effects on endocrine and exocrine secretion, angiogenesis, and cellular proliferation, primarily through interactions with SSTRs. Among the five subtypes of SSTRs, SSTR2 is the most frequently expressed in NETs (11, 12). In the present study, SSTR2 expression was observed in 65.7% of patients with multiple RNETs, with no discordant SSTR2 expression detected between the primary tumor and synchronous lesions. However, in the matched set, we found that the SSTR2 positivity rate was higher in solitary RNETs (84.4%) than in multiple RNETs (57.1%) (*p* < 0.05). SSTR2 expression is significantly associated with favorable clinical behavior and improved overall survival in patients with RNETs. In cases of metastatic disease, SSTR2 expression may serve as a potential target for somatostatin analog therapy (13).

The expression of synaptophysin and CD56 was observed in all lesions, whereas CgA was expressed in 60.0% of patients with multiple RNETs. No differences in the expression of these markers were noted between primary and synchronous NETs. In the matched set, multiple RNETs had a high rate of CgA positivity (57.1%) compared to solitary RNETs (21.9%) (*p* < 0.05). Previous studies have reported that CgA positivity in RNETs ranges from 20.8% to 30%, which is lower than the prevalence of synaptophysin-positive cases (14–16). Overexpression of CgA is associated with a pro-tumoral effect and is linked to poorer prognosis in many NETs (16, 17). Our current study found that multiple RNETs had higher expression compared to solitary RNETs. Future research with larger sample sizes and investigation into molecular mechanisms should be conducted to clarify the correlation of this phenomenon with the treatment and prognosis of the two types of tumors.

During a median follow-up of 20 months, regional LNM was observed in two patients (13.3%), both of whom experienced local recurrence and metachronous lesions. Additionally, distant metastases occurred in one patient (6.7%), while another patient (6.7%) developed only metachronous lesions. Notably, the tumor sizes in patients with local recurrence or metachronous lesions were all less than 10 mm, except for the patient with distant metastases, who had one lesion larger than 10 mm. Recurrence of NETs smaller than 10 mm has been documented in several previous studies (18). Numerous studies have demonstrated that the number of RNETs is associated with LNM (3, 5, 19). Specifically, multiple RNETs smaller than 10 mm exhibit a higher incidence of LNM (10%–22.7%) compared to solitary tumors of similar size (20). Therefore, the number of RNETs appears to be correlated with LNM, independent of tumor size.

Given the elevated risk of LNM in multiple NETs, their surveillance and treatment strategies should differ from those employed for solitary rectal NETs (20). Endoscopic resection techniques, including endoscopic mucosal resection and endoscopic submucosal dissection, are recommended for NETs measuring less than 10 mm. However, the submucosal nature of RNETs often results in incomplete endoscopic resection. Several studies have reported good complete resection rates and short-term results after the endoscopic resection of solitary RNETs (21–23) and multiple RNETs smaller than 10 mm (24, 25). The Rx and R1 resection rates in this study are relatively high at 31.4%, which may not be consistent with the previous studies. The possible reasons are as follows: first, the definition of Rx resection for rectal neuroendocrine tumors varies across different literature. In this study, the definition of Rx resection is relatively broad, including cases where the resection margin is close to the tumor, which may account for the relatively high Rx resection rate observed. Second, in our study, the sample size of multiple RNETs was relatively small. Further studies with larger sample sizes on endoscopic treatment and follow-up for this type of disease are still needed. Third, there is also significant variation in lesion size and number in previous works of literature, making it difficult to reach a consensus on the management strategy for this type of disease. Currently, there is still controversy regarding the treatment strategies for multiple rectal neuroendocrine tumors. Therefore, large-sample prospective studies are still needed in the future to verify the optimal treatment strategies for tumors with malignant potential, such as comparative studies between transanal endoscopic microsurgery and endoscopic treatment (26). There are currently no standardized guidelines for the surveillance of multiple RNETs. Nevertheless, for patients with multiple RNETs, more frequent and continuous endoscopic monitoring may be warranted to prevent the oversight of residual NET lesions and to detect local recurrences.

This study has some limitations. First, the study represents the limited experiences of a single center, and the number of cases with multiple RNETs was relatively small. Second, as a retrospective study, selection bias may have influenced the choice of treatment procedures. Therefore, a large prospective randomized controlled trial is needed to investigate critical characteristics of multiple RNETs. Finally, this study had a high loss to follow-up rate and a short follow-up period. However, due to the slow growth of RNETs, it is difficult to evaluate the long-term recurrence or survival outcomes after endoscopic resection (5). As a result, the study did not identify the factors associated with the survival of patients with multiple NETs, an issue that should also be investigated in future studies.

In conclusion, through our small-sample study, multiple RNETs demonstrated a high degree of consistency in core diagnostic indicators. Although large-scale studies are still needed for validation, this finding may potentially pave the way for reducing extensive special staining for each lesion in multiple RNETs in the future. However, compared to solitary RNETs, multifocal presentations exhibit a significantly higher propensity for metastasis/recurrence, warranting intensified therapeutic protocols and enhanced clinicopathological surveillance paradigms.

## Data Availability

The raw data supporting the conclusions of this article will be made available by the authors, without undue reservation.
